# Generating Synthetic Electronic Health Record Data Using Generative Adversarial Networks: Tutorial

**DOI:** 10.2196/52615

**Published:** 2024-04-22

**Authors:** Chao Yan, Ziqi Zhang, Steve Nyemba, Zhuohang Li

**Affiliations:** 1 Department of Biomedical Informatics Vanderbilt University Medical Center Nashville, TN United States; 2 Department of Computer Science Vanderbilt University Nashville, TN United States

**Keywords:** synthetic data generation, electronic health record, generative neural networks, tutorial

## Abstract

Synthetic electronic health record (EHR) data generation has been increasingly recognized as an important solution to expand the accessibility and maximize the value of private health data on a large scale. Recent advances in machine learning have facilitated more accurate modeling for complex and high-dimensional data, thereby greatly enhancing the data quality of synthetic EHR data. Among various approaches, generative adversarial networks (GANs) have become the main technical path in the literature due to their ability to capture the statistical characteristics of real data. However, there is a scarcity of detailed guidance within the domain regarding the development procedures of synthetic EHR data. The objective of this tutorial is to present a transparent and reproducible process for generating structured synthetic EHR data using a publicly accessible EHR data set as an example. We cover the topics of GAN architecture, EHR data types and representation, data preprocessing, GAN training, synthetic data generation and postprocessing, and data quality evaluation. We conclude this tutorial by discussing multiple important issues and future opportunities in this domain. The source code of the entire process has been made publicly available.

## Introduction

Generating synthetic versions of private human-generated data sets has garnered increasing attention in both academia and industry as a means to enable broad data access on a large scale [1,2]. When appropriately generated, synthetic data can mirror the statistical structures of the real data upon which they are based while severing connections to real human individuals [3]. Synthetic data not only enable data sharing with minimal privacy risks but also support data augmentation (ie, artificially increase the amount of available data by generating new data) to boost the performance of machine learning (ML) models. Such a nature has significant implications for maximizing the value of patient data to improve biomedicine and health care.

The widespread adoption of electronic health record (EHR) systems has amassed vast patient data globally. Despite their potential to enrich health knowledge and support care optimization [4-7], data accessibility remains limited due to privacy concerns [8,9], which impedes the advancement of knowledge discovery and translational artificial intelligence (AI) or ML research in health care. Synthetic data generation emerges as a solution by producing EHRs that are of minimal privacy risks while maintaining usability to facilitate endeavors [10,11] ranging from health information system (or software) testing and medical education to hypothesis generation and medical AI development. Acknowledging their benefits, multiple initiatives have relied upon synthetic data to expand the accessibility of their data for public use, including the National Institute of Health’s National COVID Cohort Collaborative [12] and the Clinical Practice Research Datalink by the United Kingdom’s National Institute for Health and Care Research [13].

Due in part to the limited accessibility of real EHRs, the data sets made available for biomedical research often exhibit small sizes, insufficient diversity, missing modalities, biased subpopulation representativeness, imbalanced labels, and scarce annotations [14]. As a result, ML models trained on these data may demonstrate inferior performance, limited generalizability, and unfair outcomes (ie, when there exist disparities in model performance across patient subpopulations) [15]. Compared with solely using existing data, integrating synthetic EHR data with real data can potentially enhance model performance and reduce biases [3,16,17]. This strategy effectively enlarges the proportion of underrepresented classes or patient subpopulations within the real data and, thus, prevents the model training process from overly focusing on the dominant groups. Importantly, synthetic EHR data can be produced quickly, of arbitrary size, and at low cost, and they are able to introduce higher diversity than traditional augmentation strategies (eg, over- or undersampling), which reduces the likelihood of overfitting. It is notable that creating synthetic EHR data, when based on a private real data set and supplied to support ML innovations by a third party, offers a unique opportunity to realize the dual benefits of data sharing that maintains privacy and data augmentation.

Among numerous synthetic data generation techniques, generative adversarial networks (GANs) and their variants have showcased their capability to accurately capture the statistical properties of real EHR data while inducing low privacy risks [18-20]. GAN-based methods avoid explicitly modeling clinical knowledge and making assumptions about variables and their correlations; instead, they directly learn the underlying relationships from the multidimensional data and then generate synthetic records based on the learned model [21].

Despite the rapid advancement and evolution of synthetic EHR data generation technologies, the whole procedure for producing synthetic EHR data has not been revealed in a detailed manner. This tutorial paper aims to fill that gap by providing a sequence of step-by-step instructions, supported by complementary demo code, to assist those practitioners who are not specialized in this area to effectively translate state-of-the-art research in synthetic EHR data to practical applications. This tutorial is designed with the expectation that readers have a basic understanding of ML concepts and proficiency in Python programming. We cover multiple topics, including GAN architecture, EHR data types and matrix representation, data preprocessing, GAN training, synthetic data generation, and evaluation. For demonstration purposes, we use the state-of-the-art open-source model (ie, EMR-WGAN [22]) and a publicly available EHR data set (ie, the Medical Information Mart for Intensive Care, the Fourth Version [MIMIC-IV] [23]) for structured EHR data generation. We defer the comparisons of various GAN-based models to our previous paper [21]. We also provide a detailed Jupyter notebook [24] to ensure the replicability of the tutorial content.

## Methods

### Data Set

We use the MIMIC-IV [23] data set as an example to demonstrate the generation and evaluation process of synthetic structured EHR data. MIMIC-IV is the latest version of the MIMIC EHR data, a publicly available database sourced from real EHRs of the Beth Israel Deaconess Medical Center. Adult patients admitted to the emergency department or an intensive care unit between 2008 and 2019 were incorporated. MIMIC-IV includes a wide array of information such as diagnoses, procedures, treatments, measurements, orders, free-text clinical notes, and mortality labels that indicate whether a patient died within 1 year following their last hospital stay within the timeframe. In this tutorial, we extracted patients from MIMIC-IV who had at least 1 hospital admission and were discharged alive following their last hospitalization. To build a simple demonstration data set, we extracted patients’ demographic information (including age, sex, and race); diagnoses; and 2 types of the latest measurements, that is, BMI and blood pressure (systolic and diastolic pressures). We reduced the dimensionality by converting the *International Classification of Disease, Ninth or Tenth Revision* (*ICD-9/10*) diagnosis codes to phenome-wide association study codes (ie, phecodes), which aggregate billing codes into clinically meaningful phenotypes [25].

### GAN Architecture

**Figure 1:**
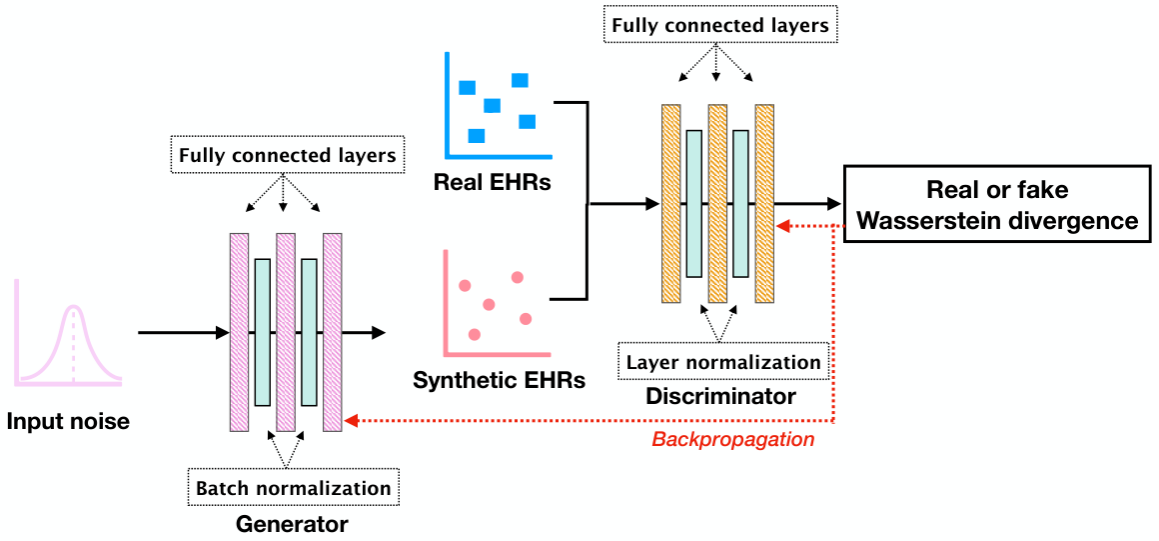
An architectural overview of EMR-WGAN. EHR: electronic health record.

GANs consist of 2 neural networks: a generator that is trained to produce realistic synthetic data from random noise and a discriminator that aims to distinguish between real and synthetic data generated by the generator [26]. During the iterative training process, the generator receives feedback through backpropagation from the discriminator and then continues to refine its capability until the discriminator cannot differentiate between real and synthetic data. GAN variants retain this common architecture while customizing how each component is implemented to adapt to various data types and stabilize the training procedure [27]. Specifically, EMR-WGAN [22] (Figure 1) applies Wasserstein divergence [28] to characterize the distance between real and synthetic data and uses fully connected layers, as well as normalization techniques, to construct the generator and discriminator. This combination of design has demonstrated its superiority in capturing the statistical characteristics of real data over other models for EHR data generation [21].

### EHR Data Types and Matrix Representation

**Figure 2:**
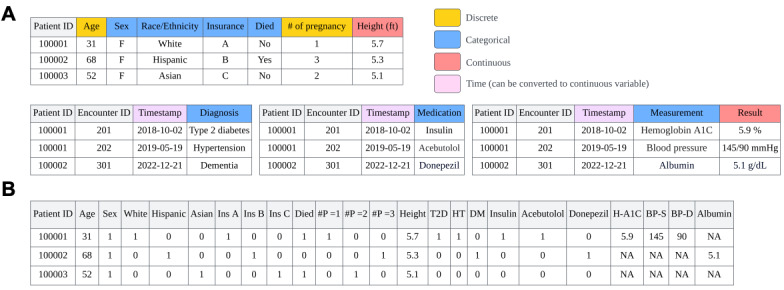
An illustration of (A) data types in electronic health record data, and (B) transformed snapshot electronic health record matrix for synthetic data generation. #P: number of pregnancies; BP-D: diastolic blood pressure; BP-S: systolic blood pressure; H-A1C: hemoglobin A1C; HT: hypertension; Ins: insurance; T2D: type 2 diabetes.

Structured EHR data for secondary analysis are usually stored in a relational database (eg, Epic Clarity) or in multiple separated files with a tabular format (eg, MIMIC-IV), where each row represents a patient’s fact, such as demographic information, or a medical event marked by a timestamp, such as disease diagnoses, medication prescriptions, measurements, medical procedures, and clinical outcomes related to an encounter. These data are usually represented by continuous, categorical, or discrete variables (Figure 2A). Continuous variables can assume any value within a specific range, making them suitable for representing medical measurement results, such as hemoglobin A_1c_ readings. Discrete variables are characterized by a countable number of numerical values, such as the number of pregnancies. However, the discrete variables with a broad range of values, such as age, can be approximated as continuous variables. In contrast, categorical variables are defined by a limited and typically unchanging set of options, such as sex, race, and diagnosis. Unlike discrete variables that naturally possess an order, categorical variables typically do not have a hierarchical order among their options, or they may display only a nominal relationship with nonquantitative distinctions, such as classifications of “low,” “medium,” or “high.” In the practice of synthetic data generation, discrete variables with a limited range of values are sometimes considered categorical for simplicity.

Timestamps indicate medical events’ positions on the time dimension. In the longitudinal synthetic EHR generation scenario, the time interval between 2 consecutive medical events is often used as a substitute for timestamps [29,30]. In this paper, we focus on demonstrating the generation of snapshot (or static) EHR data by removing or transforming the occurrence time of medical events so that all information about 1 patient can be represented by 1 single row of a table. While temporal information on medical events adds significant value to EHR data, snapshot EHR data still offers a wealth of information to support care delivery, data analytics, research, policy making, and education. Figure 2B shows a transformed snapshot EHR data matrix (EHR matrix for short) derived from Figure 2A. In this matrix, each row denotes a patient’s record, and each column denotes a variable. It is notable that each categorical variable with *k* (*k*>2) distinct options is represented by *k* new variables (or columns) in a one-hot manner (eg, insurance and number of pregnancies in the example), whereas the categorical variables with only 2 options (eg, mortality in the example) are represented by a single binary column.

**Figure 3:**
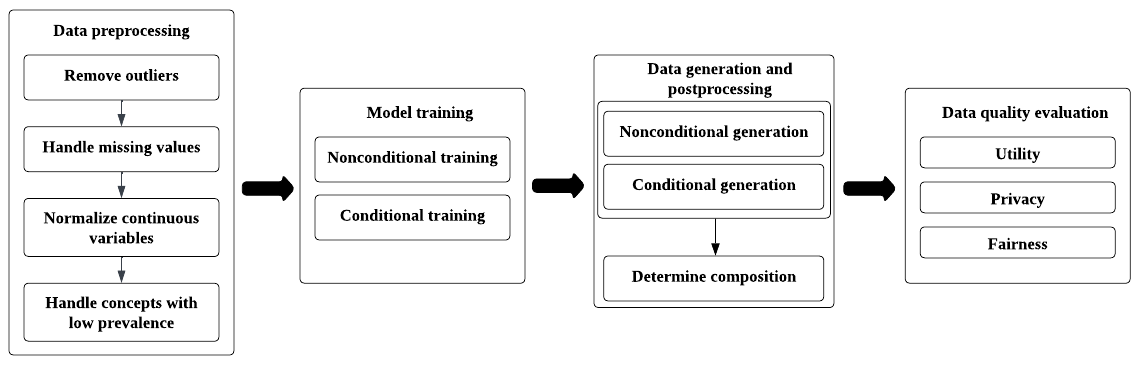
An overview of synthetic electronic health record data generation process through training generative models.

Figure 3 illustrates the whole process of producing synthetic EHR data by training generative models.

### Data Preprocessing

#### Overview

With the patient cohort of interest extracted and the corresponding matrix representation of their EHR data (ie, EHR matrix) obtained, a series of data preprocessing procedures need to be performed in order to produce a GAN-ready training data set. The procedures include (1) removing outliers, (2) handling missing values, (3) normalizing continuous variables, and (4) handling concepts with low prevalence.

#### Removing Outliers

We define outliers in structured EHR data as data points that are significantly distant from the majority of values. These can be data points that conflict with common sense or established clinical knowledge. This phenomenon typically occurs when incorrect values are entered or generated in EHRs and is particularly prevalent among discrete and continuous variables. Outliers can also represent occurrences that are theoretically possible but exceedingly rare, which creators of synthetic data may opt to exclude depending on the requirement of data generation. In both cases, it is critical to inspect the distribution of each noncategorical variable by creating histograms and reviewing basic statistical measures, such as the mean, median, minimum, and maximum values. As an example, we examined the distribution of BMIs in the processed EHR matrix, which led to findings that the minimum and maximum BMIs are 0 and 107,840.2. There are 366 patients with their latest BMIs greater than 60, and there are 120 patients with their BMIs less than 10. Given that these BMIs are unreasonable for adult patients, we removed the corresponding patients from the EHR matrix. One alternative solution that preserves the amount of data available for training generative models is to clip outlier values based on a pre-established reasonable range for the relevant variables.

#### Handling Missing Values

Multiple reasons can contribute to EHR data missingness, including, but not limited to, fragmented EHRs, incomplete documentation, data entry errors, and skipped clinical measurements. These reasons have also been classified in the literature as missing completely at random, missing at random, or missing not at random [31]. Before proceeding with imputation, it is generally recommended to eliminate variables with a high missing rate (eg, more than 50%). Numerous missing data imputation methods for EHR data have been developed [32-35], such as random sampling, prediction-based methods, and nearest neighbor–based methods. Yet, growing evidence has suggested that different methods are suitable for different missingness types, data sets, and use cases and that there is no single method that is universally considered the best for all scenarios. In this tutorial, we applied a random sampling strategy to impute missing values in BMI, which had a 38.6% missing rate, and both diastolic and systolic blood pressure, each with a 43.5% missing rate. Specifically, we randomly sampled and then imputed values based on the marginal distribution of each variable, though we acknowledge that this might not be the optimal strategy for all use cases of this data set.

#### Normalizing Continuous Variables

Continuous variables each possess a specific range of values, as illustrated by the difference between blood pressure and height in feet in Figure 2B. Normalizing continuous variables prevents the training of generative models from being dominated by variables with large ranges. To keep the distribution of each continuous variable, it is recommended to linearly compress their values into the range of (0,1), with its maximum and minimum values the same as binary variables. Given a continuous variable *v*, as well as its maximum value *v*_max_ and minimum value *v*_min_, the normalized value *v’*_k_ of *v*_k_ can be calculated as:



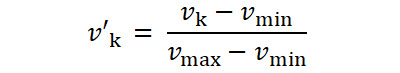



**(1)**

#### Handling Concepts With Low Prevalence

Concepts with low prevalence correspond to clinical variables that represent rare facts or events within the patient cohort. Examples include diseases, procedures, and medications that are uncommonly diagnosed, executed, and prescribed, respectively. ML-based generative models, including GANs, cannot accurately capture the statistical properties of these variables, as well as their correlations with other variables, due to the limited observations in the real data set. Noise, however, could be induced by keeping these variables in the EHR matrix for GAN training. To address this issue, several strategies can be used as follows: (1) removing these low-prevalence variables from the EHR matrix and reintroducing them in the postprocessing stage when needed, (2) rolling up variable granularity to a higher level to raise prevalence (eg, converting raw *ICD-9/10* codes to their integer level or to phecodes), and (3) combining both approaches. In this tutorial, we converted *ICD-9/10* diagnosis codes to phecodes and then removed the phecodes with a prevalence of less than 5×10^–5^.

### Model Training

Depending on model architectures, distance measures, and training techniques used (such as batch sizes, and alternating strategies for training the generator and discriminator), GAN-based synthetic EHR data generation models show varied capabilities in capturing the properties of real data. However, they typically encounter 2 main types of uncertainties throughout the training process. First, GAN training usually occurs within a parameter space that is both complex and high-dimensional. This inherent complexity and the adversarial dynamics of GANs often lead to an unstable training process that converges to suboptimal solutions. Such nature of GAN training can cause multiple undesired phenomena, including mode collapse (the generator maps different inputs to the same output) and mode drop (the generator only captures part of the distribution in the real data) [22]. Second, the model checkpoint that corresponds to the highest quality of the synthetic data is not necessarily the one with the lowest training loss. In addition, it has been realized that overtraining GAN-based models might degrade the quality of synthetic data. In other words, there is no monotonic relationship between training loss and the quality of synthetic data.

In order to attain the synthetic EHR data of the highest possible data quality that a GAN-based model can achieve, we highly recommend training the model multiple times (or multiple runs) from scratch and testing data quality at multiple checkpoints along the training trajectory of each run. This mechanism will not only improve the quality of synthetic EHR data to better support downstream uses but also contribute to more fair comparison between different generative models. This is crucial because researchers often need to select the best synthetic EHR generation model tailored to the real data sets and designated use cases [21].

Two different training paradigms can be considered for scenarios involving patient labels, for example, health outcomes (eg, mortality, readmission, and discharge), medical events of interest (eg, the presence of phenotypes and interventions), and patients’ demographic information (eg, race, sex, and age groups). The nonconditional training paradigm does not distinguish the label variables in the EHR matrix from the remaining variables, whereas the conditional training paradigm uses the label variables to guide model training, as well as the generation of the synthetic EHR data [22], which enables the control over the categories of the generated data in terms of the label variables. Conditional training is usually achieved by incorporating the label variables as extra input of the neural networks of the generator and discriminator. However, consensus has not been established regarding which paradigm achieves a higher quality of synthetic EHR data.

When categorical variables with *k* (*k*>2) unique options are converted into *k* binary variables within the EHR matrix, it is essential to maintain the one-hot constraint in the synthetic data. This means that only 1 of the binary variables can take a value of 1, while the remaining *k*–1 variables must be set to 0. However, the GAN training mechanism may lead to a violation of this constraint. To solve this issue, a SoftMax layer should be attached to the output of the generator to preserve the one-hot constraint.

Additionally, real data may contain critical record-level constraints that represent established clinical knowledge, which need to be preserved in the synthetic data. For instance, female patients should not be assigned male-specific diseases, such as prostate cancer. Such constraints can be effectively enforced by adding corresponding penalty terms to the loss function of GANs [36].

In this tutorial, for illustrative purposes, we use the nonconditional paradigm, preserve the one-hot constraints, yet refrain from imposing record-level constraints during model training to showcase the phenomenon of clinical knowledge violation in results.

### Synthetic Data Generation and Postprocessing

Random noises, typically drawn from the standard normal distribution, need to be input into the trained generator to produce synthetic EHR data. By repeating this process, the generator is able to produce a specified quantity of synthetic records. When the conditional training paradigm is adopted, the prespecified label values should also be fed into the generator as part of the input. The capability to generate synthetic data in any desired quantity and to control the categories of the generated records affords us the flexibility to determine the composition of the resultant data set for downstream use. This nature has significant implications for data augmentation as it enables practitioners to augment their existing data sets with synthetic records tailored to their specific needs.

By applying a sigmoid or SoftMax function as the output layer of the generator, variables in the synthetic data assume values ranging between 0 and 1. For noncontinuous variables, rounding the values is necessary, whereas the values of continuous variables require rescaling to their original range by applying the inverse version of Equation 1. This process ensures that the synthetic data preserves the value ranges found in the real data set.

### Data Quality Evaluation

#### Overview

The quality evaluation of synthetic EHR data primarily revolves around 3 key aspects: data utility, privacy, and fairness. This process requires a comparison between synthetic data and real data using a set of metrics. In this tutorial, we select multiple commonly used metrics that are complementary to each other to demonstrate data evaluation. Below, we provide a brief overview of these metrics. For more comprehensive details, we point readers to several recent publications in the field [18,19,21], which provide in-depth explanations of how these metrics are designed.

Data utility measures the usefulness and applicability of a data set for specific purposes. More concretely, it is evaluated by determining how well the generated data captures the critical characteristics present in the real EHR data. Unlike imaging data whose quality can be visually evaluated by humans or assessed using a single metric, the quality of synthetic EHR data is less intuitive and can vary in a variety of aspects. Typically, data utility is assessed by evaluating the extent to which synthetic EHR data (1) resemble the statistical characteristics of real data at both variable and record (or patient) levels and (2) retain the capability of developing ML models that perform comparably to those trained using real data. In earlier research, the concept of resemblance was often characterized as being distinct and independent from data utility. Variable-level characteristics include but are not limited to, variables’ marginal distributions, their correlations, and joint distributions, whereas record-level characteristics cover multiple crucial aspects, including the violation rate of clinical knowledge, the distribution of medical concept quantity, etc.

#### Dimension-Wise Distribution

This metric evaluates the degree to which a synthetic data set captures the marginal distributions of variables in the real data. It calculates the average of the absolute prevalence differences (APDs) for categorical variables and the average of the Wasserstein distances for continuous variables between real and synthetic data sets. When both types of variables are present, we add these 2 values together and then normalize the sum to derive the final score, which is referred to as dimension-wise distance (DWD). A lower value of this metric indicates a higher level of data utility.

#### Column-Wise Correlation

This metric measures how well a synthetic data set maintains the correlations of variables present in the real data. It calculates the Pearson correlation coefficient matrices (for all variable pairs) in both the real and synthetic data sets and then computes the average of the absolute differences between corresponding cells in these 2 matrices. A lower value of this metric indicates a higher level of data utility.

#### Latent Cluster Analysis

This metric evaluates the effectiveness of a synthetic data set in preserving the underlying structures (or joint distribution) of real data in the latent space. It involves combining the real and synthetic EHR matrices and then applying principal component analysis to project the combined data set into a latent space that covers a specific threshold of variance in the system. Subsequently, a clustering algorithm, such as *k*-means, is used to derive the latent deviation, which is calculated as the logarithmic average of the transformed ratio of real data points present in each identified cluster. A lower value of this metric suggests a closer resemblance of the synthetic data set’s latent distribution to that of the real data.

#### Medical Concept Abundance

This metric quantifies the degree to which a synthetic data set maintains the quantity of the record-level information in the real data. The normalized Manhattan distance between the histograms of the number of distinct record-level medical concepts for real and synthetic data sets is calculated as the medical concept abundance distance. A lower value of this metric indicates a higher level of real-synthetic data similarity.

#### Clinical Knowledge Violation

This metric measures the degree to which a synthetic EHR data set violates clinical knowledge, particularly in terms of maintaining record-level consistency with established medical common sense. To do so, we identified the most prevalent diagnoses (3 in this tutorial) that are only associated with 1 sex in the real data and subsequently computed the average ratio of all diagnoses appearing in the opposite sex in the synthetic data sets. A lower value of this metric indicates a higher level of data utility.

#### Prediction Performance

This metric evaluates the capability of a synthetic EHR data set to support ML model development. The real data set is split into a training set and a testing set. The reference model is then trained using the real training set and evaluated on the real testing set by calculating the area under the receiver operating characteristic curve (AUROC). Subsequently, a new model is trained using the synthetic data set and then evaluated on the same real testing set. These 2 scenarios are referred to as training on real testing on real (TRTR) and training on synthetic testing on real (TSTR), respectively. The more closely the AUROC of TSTR aligns with that of TRTR, the higher the utility of the synthetic data set.

#### Feature Importance

This metric focuses on assessing how reliably a synthetic data set reveals key features that are significant in the prediction task. We first identified the top *N* (20 in this tutorial) important features in the TRTR scenario by computing the Shapley additive explanations values of all features and then computed the overlap proportion of the top *N* features with those identified in the TSTR scenario. The higher the proportion, the higher the data utility. Note that “feature” used in the context of feature importance is equivalent to variable.

Data privacy evaluation is crucial when considering the sharing of synthetic EHR data. While synthetic EHR data are designed to minimize privacy risks by severing the linkage to real patients, it is still important to conduct thorough privacy evaluations to ensure the preservation of individual privacy in multiple privacy inference settings, where adversaries’ knowledge and objectives differ. Across different privacy inference settings, it is commonly assumed that adversaries only have access to the generated synthetic data, but not the synthetic data generation model. Examples of widely used privacy metrics include membership inference risk and attribute inference risk [21,22,37], each with values ranging from 0 to 1. Membership inference risk measures the ability of an adversary to infer whether a specific real record is part of the data set to train the synthetic data generation model. It is quantified using the *F*_1_-score of the inference based on the distances between targeted records and all synthetic records. By contrast, attribute inference risk reflects an adversary’s capability to infer sensitive attributes of partially observed real EHRs. Specifically, it is calculated through the weighted sum of *F*_1_-scores of the inferences against sensitive attributes.

Multiple additional metrics have been created to assess privacy risks in various contexts, including meaningful identity disclosure risk [38] and nearest neighbor adversarial accuracy risk [39]. Meaningful identity disclosure risk extends the concept of identity disclosure from the context of releasing real data to the scenario of sharing synthetic data. It encompasses a comprehensive privacy risk that involves two main aspects: (1) inferring the identifiability of patients and (2) acquiring new knowledge about targeted patients. In contrast, nearest neighbor adversarial accuracy risk assesses the extent to which a synthetic data set overfits the real training data set. Specifically, it measures the difference between (1) the aggregated distance between synthetic records and those in the real testing data set and (2) the aggregated distance between synthetic records and those in the real training data set.

Synthetic EHR data are also anticipated to fairly represent patient subpopulations with respect to protected attributes, such as age groups, sex, race, and ethnicity. Distributional differences or distances between real and synthetic data with respect to the protected attributes of interest are often used as metrics to evaluate fair representation [40]. To ensure fair data quality, synthetic data may need to show similar variations in preserving data utility and protecting privacy for each patient subpopulation, akin to their real data counterparts. This consideration of fairness requires that utility and privacy evaluations of synthetic data should be performed independently within each subpopulation and then compared across them. Another fairness consideration necessitates that synthetic data sets provide equal support for downstream AI or ML tasks across all subpopulations, regardless of the basis of the real data. Due to the complexity surrounding fairness and the absence of clear guidelines for evaluating it in synthetic EHR data, we will skip this evaluation in our demonstration.

It is crucial to note that quality evaluation of synthetic EHR data should be tailored to align with specific use cases because different use cases prioritize the preservation of different data aspects. For instance, when the synthetic EHR data are intended to facilitate hypothesis generation to support medical research in a controlled research environment, the evaluation would emphasize metrics that measure disease prevalence and correlations between features and outcomes, while privacy risks may be of lesser concern. On the other hand, if the synthetic EHR data are developed to support the development of clinical decision support software by third-party developers, evaluating privacy risks becomes more critical than determining whether the synthetic data preserves the nuanced statistical properties of the real data. Our previous research provides a use case-oriented benchmarking framework to enable systematic comparisons of synthetic data generation models [21]. The users of this framework determine the prioritization of evaluation metrics by providing a weight profile, which applies to the evaluation results from individual metrics and represents the relative importance or preference assigned to each metric. The final score of a synthetic data set or a synthetic data generation model is derived by aggregating the weighted results for all considered metrics.

Using this benchmarking framework enables the selection of the most suitable synthetic data set for a specific use case or the comparison of various synthetic data generation models (not necessarily limited to those that are GAN-based) based on the scores assigned to produced synthetic data sets.

## Results

### Overview

In this section, we present the results of data quality evaluation for synthetic EHR data sets in terms of data utility and privacy. Furthermore, we demonstrate how to compare these synthetic EHR data sets to identify the most suitable one for specific use cases. To do so, 70% of records of the preprocessed MIMIC-IV data set were used to train the EMR-WGAN model and the remaining 30% of records were used for evaluation purposes. Considering the inherent uncertainties associated with GAN-based model training as mentioned earlier, EMR-WGAN was independently trained 5 times. While we recommend examining multiple checkpoints during each model’s training phase, for the purposes of this demonstration, we selected an epoch with a relatively low training loss from each independent training session to generate the corresponding synthetic data set. All synthetic data sets produced by these models have the same size as the real training data set. The complete process of data quality evaluation can be found in the shared Jupyter notebook [24].

### Characteristics of the Real Data Set

Table 1 provides an overview of the basic characteristics of the MIMIC-IV cohort selected for the creation and evaluation of synthetic EHR data. We initially included a total of 181,294 patients who had at least 1 hospital admission and were discharged alive for their last hospital stays. The average age of this cohort is 56.2 (SD 20.4) years. This cohort comprises 96,617 (53.3%) female individuals and multiple racial groups, with 7667 (4.2%) Asian; 23,999 (13.2%) Black; 10,058 (5.5%) Hispanic; 121,954 (67.3%) White; 10,078 (5.6%) belonging to other races; and 7538 (4.2%) of unknown race. A total of 20,493 (11.3%) of the cohort died within 1 year after their last hospital stay. The data preprocessing procedure led to the removal of 548 patients and more reasonable distributions of BMI, diastolic, and systolic blood pressures. The curated real EHR matrix contains 1460 columns after we removed 140 extremely rare diagnoses.

**Table 1:** Cohort characteristics before and after data preprocessing.

Characteristics			Distributions and values		
			Before preprocessing (n=181,294)		After preprocessing (n=180,746)
Cohort size, n (%)			181,294 (100)		180,746 (100)
Age (y), mean (SD)			56.2 (20.4)		56.2 (20.3)
**Sex, n (%)**					
	Female	96,617 (53.3)		96,304 (53.3)	
	Male	84,677 (46.7)		84,442 (46.7)	
**Race, n (%)**					
	Asian	7667 (4.2)		7654 (4.2)	
	Black	23,999 (13.2)		23,889 (13.2)	
	Hispanic	10,058 (5.5)		10,035 (5.6)	
	White	121,954 (67.3)		121,603 (67.3)	
	Others	10,078 (5.6)		10,049 (5.6)	
	Unknown	7538 (4.2)		7516 (4.2)	
Died within 1 year, n (%)			20,493 (11.3)		20,414 (11.3)
BMI, mean (SD)			21.1 (277.03)		28.4 (6.8)
Diastolic blood pressure, mean (SD)			47.6 (36.4)		73.6 (11.8)
Systolic blood pressure, mean (SD)			81.9 (62.3)		126.6 (18.2)
**Top 10 prevalent diagnoses (in phecodes), n (%)**					
	Hypertension (401)	57,238 (31.6)		57,056 (31.6)	
	Disorders of lipoid metabolism (272)	39,216 (21.6)		39,103 (21.6)	
	Other anemias (285)	33,979 (18.7)		33,844 (18.7)	
	Essential hypertension (401.1)	31,694 (17.5)		31,541 (17.5)	
	Hyperlipidemia (272.1)	28,011 (15.5)		27,896 (15.4)	
	Diseases of esophagus (530)	25,887 (14.3)		25,800 (14.3)	
	Cardiac dysrhythmias (427)	25,284 (14)		25,195 (13.9)	
	Mood disorders (296)	25,201 (13.9)		25,089 (13.9)	
	Tobacco use disorder (318)	24,152 (13.3)		24,054 (13.3)	
	Disorders of fluid, electrolyte, and acid-base balance (276)	23,895 (13.2)		23,807 (13.2)	
	Diabetes mellitus (250)	23,789 (13.1)		23,695 (13.1)	
Total number of columns in electronic health record matrix			1600		1460

### Data Utility

**Figure 4:**
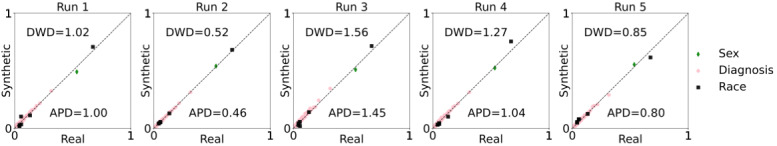
Dimension-wise distribution for categorical variables. The dashed diagonal line indicates the perfect replication of variable prevalence. APD: absolute prevalence difference; DWD: dimension-wise distance.

Figure 4 illustrates the dimension-wise distribution results and the associated APD for categorical variables. Although all 5 runs effectively maintain the marginal distributions of these variables, the second run exhibits the smallest APD. When considering both the categorical and continuous variables (ie, age, BMI, diastolic, and systolic blood pressures), the second run still achieves the lowest DWD. By contrast, the third run is associated with the highest DWD, indicating a relatively low effectiveness in preserving dimension-wise distributions.

**Figure 5:**
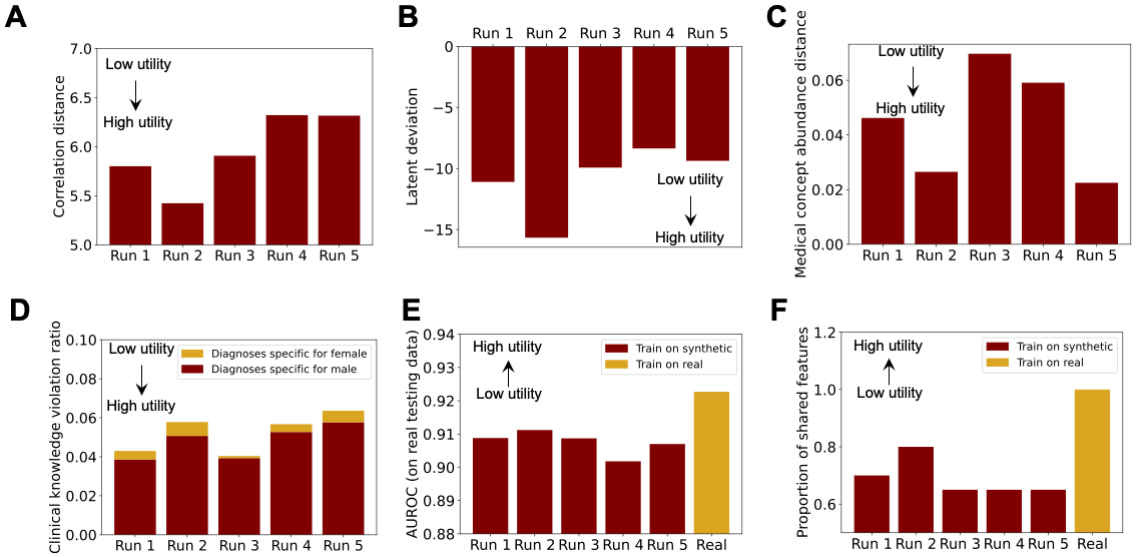
Data utility in (A) column-wise correlation, (B) latent cluster analysis, (C) medical concept abundance, (D) clinical knowledge violation, (E) prediction performance, and (F) feature importance. For clinical knowledge violation, “hyperplasia of prostate," “cancer of prostate,” and “erectile dysfunction” are examined as male-specific diagnoses (in phecodes); “other conditions or status of the mother complicating pregnancy, childbirth, or the puerperium,” “known or suspected fetal abnormality affecting management of mother,” and “other complications of pregnancy necrotizing enterocolitis” are examined as female-specific diagnoses (in phecodes). AUROC: area under the receiver operating characteristic curve.

Figure 5 summarizes the evaluation results of the 5 synthetic data sets for the remaining 6 data utility metrics, with the indication of directional implications of the values under each metric. Notably, the second run demonstrates the highest data utility in column-wise correlation, latent cluster analysis, prediction performance, and feature importance and secures the second position in medical concept abundance. Yet, its score in clinical knowledge violation is positioned fourth. Additionally, it was observed that male-specific diagnoses are more than 10 times as likely to be incorrectly assigned to female records in the synthetic data sets compared with similar violations for female-specific diagnoses. This suggests that the correlations between sex and sex-specific diagnosis columns were not equally preserved, possibly resulting from different levels of complexity (or noise) in the data pertaining to different sexes. While this phenomenon falls beyond the scope of this tutorial, it merits further exploration.

### Privacy

Table 2 presents the privacy risk associated with each synthetic EHR data set in terms of membership inference attack and attribute inference attack. It also includes a baseline comparison, which corresponds to an extreme situation of releasing real data. Compared with the real data set, every synthetic data set achieves substantially reduced risks. While the variance in risk levels among the 5 synthetic data sets is relatively small, the second run exhibits the highest membership inference risk and the second lowest risk in attribute inference.

**Table 2:** Privacy risks of synthetic electronic health record data sets. For each risk category, the identical risk value is attributed to a loss of precision.

Risk type	Run 1	Run 2	Run 3	Run 4	Run 5	Real
Membership inference	0.29	0.31	0.29	0.29	0.30	0.91
Attribute inference	0.14	0.14	0.14	0.13	0.14	0.97

### Identifying the Most Suitable Synthetic Data Set for a Specific Use Case

We have obtained the evaluation results of all 5 synthetic data sets for individual metrics, allowing for straightforward derivation of their rankings in each metric as presented in Table 3. A smaller ranking position indicates better data quality. In this tutorial, we consider two distinct use cases of synthetic EHR data: (1) ML model development, which prioritizes the performance of prediction tasks and model explainability, and (2) education, which focuses more on the record-level consistency with clinical knowledge, prevalence of diagnoses, and privacy. We proposed example weight profiles for these 2 use cases and then calculated the overall rankings of the synthetic data sets for each scenario. The analysis identifies the second and third runs as the most suitable data sets for ML development and education, respectively. This observation further justifies that the quality evaluation of synthetic data should be in the context of use cases.

**Table 3:** Data quality rankings of synthetic data sets. Weight profiles A and B correspond to the use cases for supporting machine learning model development and education, respectively. Overall rankings of data sets are weighted summation of individual rankings in all metrics.

Metric		Weight profile A	Weight profile B	Run 1	Run 2	Run 3	Run 4	Run 5
**Utility**								
	Dimension-wise distribution	0.1	0.1	3	1	5	4	2
	Column-wise correlation	0.1	0.1	2	1	3	5	4
	Latent cluster analysis	0.1	0.0	2	1	3	5	4
	Medical concept abundance	0.0	0.0	3	2	5	4	1
	Clinical knowledge violation	0.1	0.4	2	4	1	3	5
	Prediction performance	0.2	0.0	2	1	3	5	4
	Feature importance	0.2	0.0	2	1	4	4	4
**Privacy**								
	Membership inference	0.1	0.2	3	5	2	1	4
	Attribute inference	0.1	0.2	3	2	4	1	5
Overall rankings for weight profile A		N/A^a^	N/A	2.3	1.8^b^	3.2	3.7	4.0
Overall rankings for weight profile B		N/A	N/A	2.5	3.2	2.4^b^	2.5	4.4

^a^N/A: not applicable.

^b^Indicates the most suitable data set for each use case.

## Discussion

### Principal Findings

GAN-based synthetic data generation has demonstrated significant potential to enlarge the accessibility of health data and enhance the effectiveness of ML in health care [41-43]. This tutorial demonstrates how to create and evaluate structured synthetic EHR data by applying a GAN-based generative model to a publicly available EHR data set. Beyond introducing technical details, we aim to discuss several important issues related to this topic.

GAN-based synthetic EHR data generation models exhibit limited capability in accurately representing and then generating the concepts with low prevalence. This is also a common challenge for almost all ML methods. From our experience, incorporating these concepts into the real data for GAN training, compared with removing them, can result in adverse effects on capturing the distributions of prevalent concepts. In settings where accurate representation of concepts with low prevalence is crucial (eg, synthetic data are developed to replicate studies related to rare diseases), additional efforts should be dedicated to ensuring their fidelity in the synthetic data. One solution is to increase the representation of these concepts in the real data through data collection or data oversampling. The second solution is to independently model the cohort associated with the targeted concept. Subsequently, the synthetic data for this specific cohort can be generated and then merged with the main synthetic data. Another approach, which is modeling-free, is to perturb the real EHR data with the targeted concept based on expert knowledge and then add the resultant data back into the main synthetic data. It should be noted that the quality of synthetic data after using these approaches should be comprehensively evaluated.

Selecting the most suitable synthetic EHR data set or synthetic data generation model for a targeted use case is subject to 2 types of tradeoffs: extrinsic and intrinsic tradeoffs. Users of this technology control the extrinsic tradeoff by prioritizing which aspects of the data to preserve in data quality evaluation. This can be accomplished by using an appropriate set of evaluation metrics and assigning weights to each metric to achieve a balanced evaluation outcome that aligns with the use case, as mentioned earlier. Different prioritization strategies can yield variations in evaluation results, thereby influencing the selection of the optimal data set or model.

The intrinsic tradeoff arises from the inherent interrelation and tension among data utility, privacy, and fairness. In general, better data utility aligns with a more accurate representation of the nuanced statistical characteristics present in the real data, which can, in turn, improve the success rate of privacy inference regarding sensitive information about patients. Similarly, aiming for a higher level of privacy protection is often paired with a reduction in data fidelity. Different synthetic EHR generation models, and even different runs of the same model, can exhibit varying utility-privacy tradeoffs. The choices of model structures, parameter settings, data preprocessing, and learning methods can all impact the resulting tradeoff. In addition, one can integrate privacy protection strategies during model training, such as differential privacy, to induce more privacy protection. However, for the use cases that demand high fidelity of synthetic EHR data, such as data analysis or augmenting medical AI development, the integration of additional privacy safeguards may potentially limit the value of synthetic data for the intended scenarios.

Pursuing either a higher overall utility of synthetic EHR data or stronger privacy may lead to poor fairness across patient subpopulations. This is because different patient subpopulations may not be equally affected and that the unique characteristics of underrepresented groups are more likely to be neglected. Similarly, focusing solely on fairness may result in a lower level of overall data utility or privacy. As such, both extrinsic and intrinsic tradeoffs among data utility, privacy, and fairness impact the determination of the most suitable synthetic EHR data or synthetic EHR data generation model for a specific use case.

Multiple key questions regarding the best practice of synthetic EHR data generation remain unanswered in the literature. First, the determination of the appropriate size of real data needed to train GANs and other generative models for a specific data generation task, along with an effective estimation approach, is uncertain and lacks comprehensive research. Second, the scalability of GANs and other generative models with respect to varying sizes of the variable space is still not well understood. Third, the optimal matrix representations of various EHR data types, in particular when mixed together, are relatively unexplored in current research. All of these questions need to be answered through systematic research.

The evolvement of synthetic EHR data generation technology presents numerous opportunities for various applications and advancements. We conclude this paper by highlighting several future research directions that are worth exploring and summarizing the limitations of this tutorial.

Most cutting-edge approaches for structured synthetic data generation, including EHR data, rely on a matrix or tabular representation of the real data, which involves merging all information into a single table as part of data preprocessing. When addressing the emerging need to generate a synthetic version of a relational EHR database, where patients’ data are distributed in multiple tables, such as the widely adopted OMOP common data model, joining relevant tables together can lead to an unmanageable data size with significant redundancy. There is a strong need for a novel synthetic EHR data generation paradigm that can directly learn from the original database, including its structural relationships, to address the current limitations in the field.

EHR data, in a broad sense, encompass multiple modalities, including structured health information, textual notes, medical imaging data, genetic information, and more. Current synthetic EHR data generation algorithms are designed to handle a single modality at a time, leading to a lack of consistency between separately generated data when attempting to describe the same patient. Methodology innovations are required to effectively harmonize the available modalities in EHR data during model training and then generate synthetic data that cover and represent these modalities. The core objective of this task is to learn an accurate latent representation of a patient across different modalities.

Since 2023, large language models, such as OpenAI’s ChatGPT and Google’s Med-PaLM 2, have gained substantial attention due to their remarkable ability to generate high-quality free text responses to users’ questions and instructions. Such exceptional ability stems from their extensive pretraining on vast amounts of textual data, which contain a wide range of human knowledge and common sense. In addition, the users of these models can demand the desired format of their output such as CSV and JSON. This entails a new opportunity for synthetic EHR data generation. While private EHR data have not been used by these models, an appropriate fine-tuning process using real EHR data can quickly shape them into synthetic EHR data generators. Compared with other generative methods, large language models could potentially strengthen the generation of synthetic EHR data in multiple critical aspects. First, large language models have encoded complex knowledge and relationships between medical concepts through extensive pretraining. When fine-tuned on real EHR data sets, they can more easily capture the nuances in intricate patient data and understand the underlying data semantics, which would not be easily achieved by other generative models. Second, large language models can generate data with stronger contextual relevance and coherence. In other words, they are more capable of producing data that are not only syntactically and semantically correct but also consistent with real-world scenarios and knowledge. Third, with prompt-level customization, these models can be tailored to generate specific types of EHR data in a more flexible and efficient manner, significantly reducing the human effort required in postprocessing compared with previous methods.

This tutorial has several limitations. First, it focuses on simulating static structured EHR data and neglects the timestamping of medical events. However, it is important to note that EHR data inherently consists of time series, where the temporal information is critical for numerous applications, such as modeling the progression of diseases. To address this, multiple generative models have been developed to produce temporal EHR data, a process that shares similar principles to those demonstrated in this tutorial. Second, the real data set we used for demonstration purposes does not fully capture the complexity inherent in real snapshot EHR data. It is likely that a transformed snapshot EHR matrix contains a subset of columns governed by complex semantic constraints, which may not be straightforward to implement during model training. For example, a snapshot EHR matrix for a women’s health cohort may include columns indicating the age and method (nature vs cesarean) for each childbirth. This scenario compounds constraints in several aspects, including patterns of missing data (eg, the data set might not contain only a record of the second delivery), the age at each delivery (eg, ages for subsequent deliveries should be older than previous ones), and time intervals between deliveries (eg, there should be a minimum gap of 10 months between each). Addressing this type of complex constraint is still an open research question and needs more investigation.

### Conclusions

Creating synthetic EHR data has been increasingly pursued to address the limited availability of real EHR data to facilitate various endeavors in the health domain. This tutorial provides a comprehensive guide to the entire process of generating synthetic structured EHR data using GANs, ranging from data representation, preprocessing, model training, and postprocessing to data generation and evaluation. By following this tutorial, as well as the open-sourced example based on the MIMIC-IV data set, we anticipate that potential users of synthetic data generation technology can understand and implement all involved components, and then correctly evaluate the produced data sets and interpret the evaluation results to fulfill their data needs.

## Abbreviations

AI: artificial intelligence
APD: absolute prevalence difference
AUROC: area under the receiver operating characteristic curve
DWD: dimension-wise distance
EHR: electronic health record
GAN: generative adversarial network
ICD-9/10: International Classification of Disease, Ninth or Tenth Revision
MIMIC-IV: Medical Information Mart for Intensive Care, the Fourth Version
ML: machine learning
TRTR: training on real testing on real
TSTR: training on synthetic testing on real
